# Methyl α-d,l-sorboside

**DOI:** 10.1107/S2414314626008588

**Published:** 2026-08-20

**Authors:** Tomoko Takashita, Yoji Horii, Kei Takeshita, Tomohiko Ishii

**Affiliations:** a Graduate School of Science for Creative Emergence Kagawa University, 2217-20 Hayashi-cho Takamatsu Kagawa 761-0396 Japan; b Fushimi Pharmaceutical Co Ltd, 307 Minatomachi, Marugame, Kagawa 763-8605, Japan; Katholieke Universiteit Leuven, Belgium

**Keywords:** crystal structure, hydrogen bonding, rare sugar, methyl sorboside, racemic compound

## Abstract

The title compound, C_7_H_14_O_6_, was prepared from an equimolar mixture of d- and l-sorbose (d:l = 1:1) by Fischer glycosidation in methanol and crystallized from water. The title compound crystallizes in the ortho­rhom­bic space group *Pna*2_1_. The asymmetric unit contains one mol­ecule each of methyl α-d-sorboside and methyl α-l-sorboside. In the crystal, the mol­ecules are linked by O—H⋯O hydrogen bonds, forming a two-dimensional network parallel to (001).

## Abstract 2

The title compound, methyl α-d,l-sorboside, was prepared from an equimolar mixture of d- and l-sorbose (d:l = 1:1) by Fischer glycosidation in methanol. The asymmetric unit contains one mol­ecule each of methyl α-d-sorboside and methyl α-l-sorboside, which are linked by O—H⋯O hydrogen bonds into a two-dimensional network parallel to (001).

## Graphical abstract

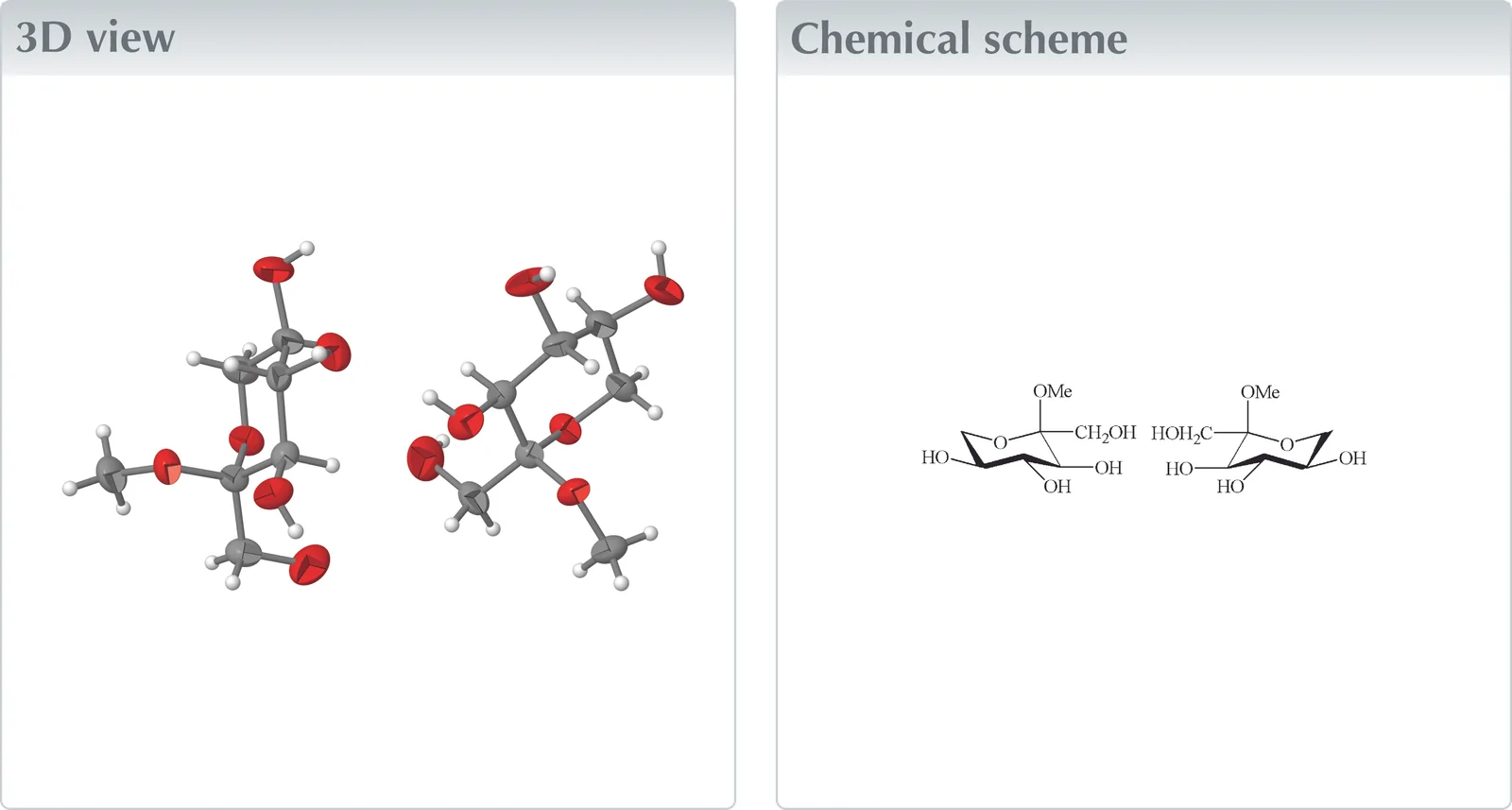

## Structure description

l-Sorbose is a rare sugar and was the first l-form hexose found in nature (Nordenson *et al.*, 1979 ▸). Sorbosides are derivatives of sorbose in which the anomeric hy­droxy group at C2 is replaced by an alk­oxy group. The crystal structures of racemic α-d,l-sorbose (CSD refcode HELYOP; Taguchi *et al.*, 2018 ▸) and methyl α-l-sorboside monohydrate (CSD refcode LAMPAU; Matsumoto *et al.*, 2021 ▸) have previously been reported. In the present study, single crystals of anhydrous methyl α-d,l-sorboside were prepared to determine its mol­ecular structure, crystal packing and inter­molecular inter­actions.

**Figure 1:**
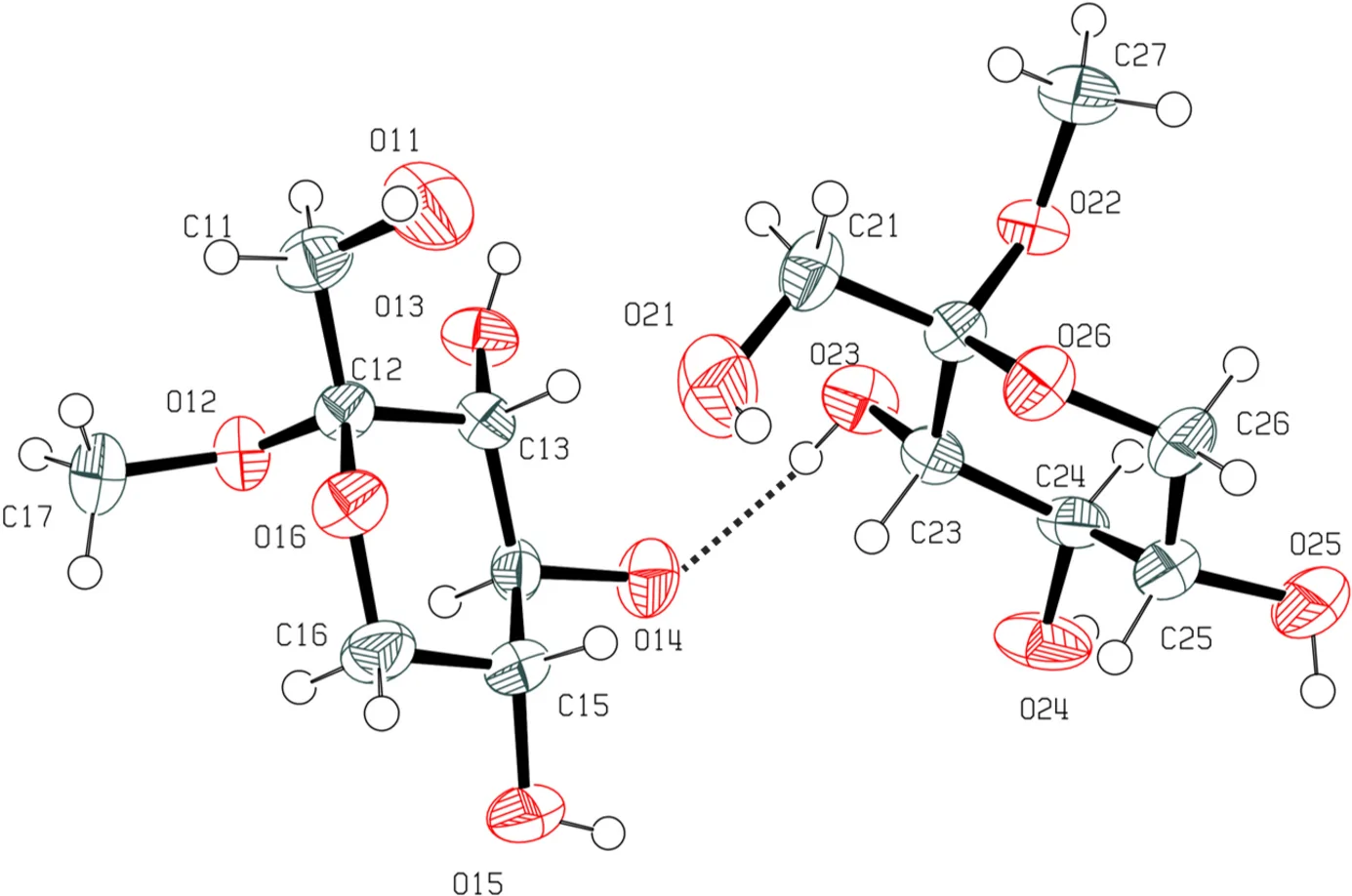
Mol­ecular structure of the two crystallographically independent mol­ecules in the asymmetric unit, showing the atom-labelling scheme. Displacement ellipsoids are drawn at the 50% probability level. The O23—H23⋯O14 hydrogen bond linking the two mol­ecules is shown as a dashed line. H atoms are shown as spheres of arbitrary radius and are not labelled.

The title compound crystallizes in the ortho­rhom­bic space group *Pna*2_1_. The asymmetric unit contains two crystallographically independent mol­ecules, one mol­ecule each of methyl α-d-sorboside and methyl α-l-sorboside (Fig. 1 ▸). The d and l enanti­omers adopt mirror-related ^5^C_2_ and ^2^C_5_ chair conformations, respectively. In both mol­ecules, the meth­oxy substituent at the anomeric carbon atom (C12 or C22) occupies an axial position. The unit cell therefore contains four mol­ecules of each enanti­omer.

The formula weight of methyl α-d,l-sorboside is 194.18, approximately 7.8% greater than that of α-d,l-sorbose (180.16). As both structures have *Z* = 8, their unit-cell volumes can be compared directly. The unit-cell volume of the title compound [1683.06 (13) Å^3^] is 232.20 Å^3^, or approximately 16.0%, larger than that of α-d,l-sorbose [1450.86 (6) Å^3^; Taguchi *et al.*, 2018 ▸]. This increase is consistent with the replacement of the anomeric hy­droxy group by a meth­oxy group, although the unit-cell volume also depends on the mol­ecular packing.

**Figure 2:**
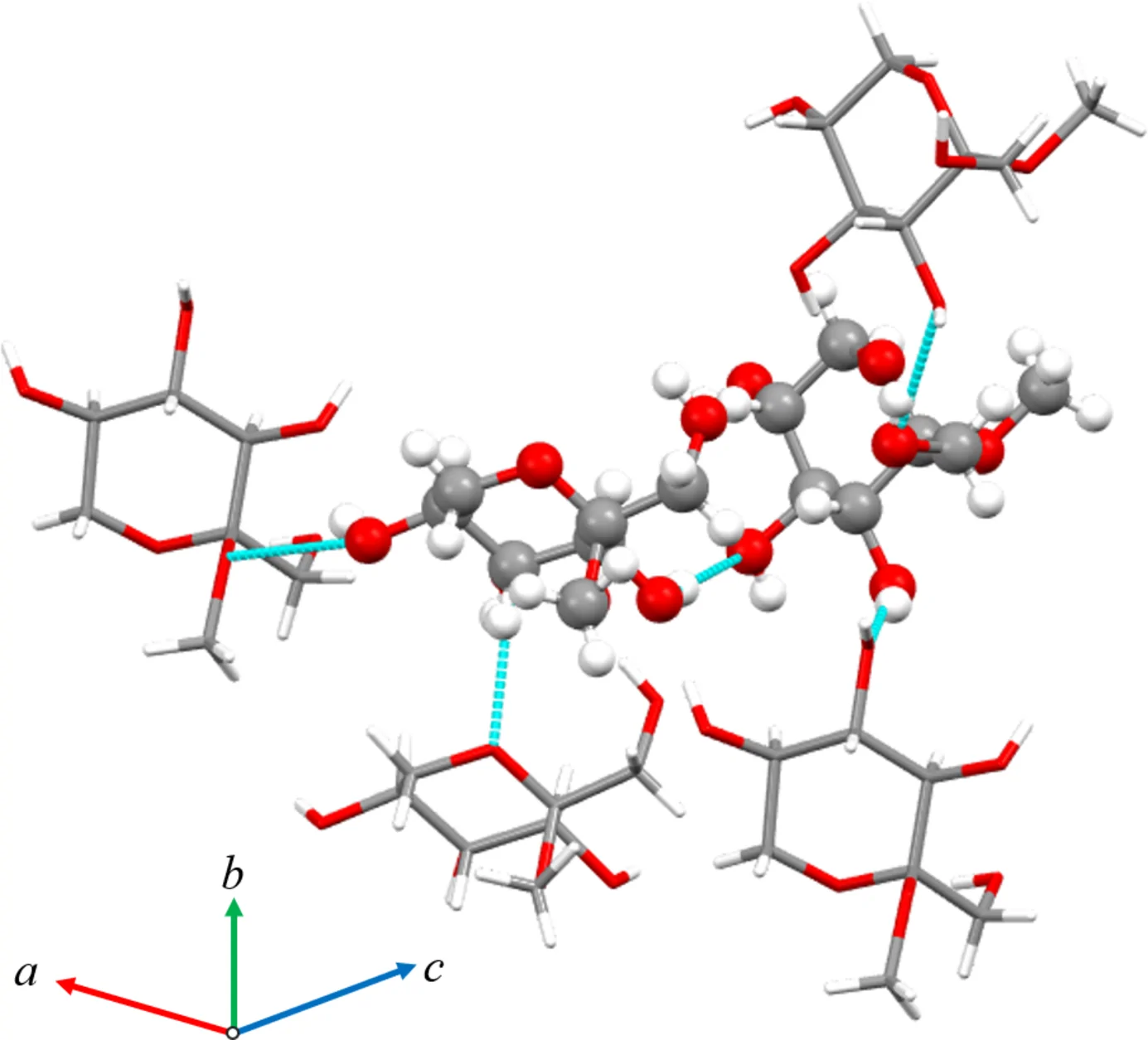
A portion of the crystal packing showing the five O—H⋯O hydrogen bonds listed in Table 1 ▸ as dashed lines. These inter­actions generate a two-dimensional hydrogen-bonded network parallel to (001). The crystallographic axes are indicated. The mol­ecules highlighted at the centre are shown using a ball-and-stick representation, whereas the surrounding mol­ecules are shown using a capped-stick representation.

In the crystal, the mol­ecules are linked by the five inter­molecular O—H⋯O hydrogen bonds listed in Table 1 ▸: O23—H23⋯O14, O24—H24⋯O26, O25—H25⋯O22, O11—H11⋯O13 and O13—H13⋯O24. The donor⋯acceptor distances range from 2.748 (4) to 2.924 (4) Å. The meth­oxy oxygen atom O22 and the ring oxygen atom O26 act as acceptors, while the remaining listed acceptors are hy­droxy oxygen atoms. Collectively, these hydrogen bonds generate a two-dimensional network parallel to (001), as shown in Fig. 2 ▸.

## Synthesis and crystallization

Methyl α-d,l-sorboside was prepared from an equimolar mixture of d- and l-sorbose (d:l = 1:1) by Fischer glycosidation in methanol. The reaction product was purified by ion-exchange chromatography using Dowex 50 W–X2 resin in the Ca^2+^ form, with deionized water as the eluent. Fractions containing the target compound, as identified by HPLC, were combined and concentrated to give a syrup. The product was then dissolved in water, and the solution was allowed to evaporate slowly at room temperature. Colorless block-shaped single crystals suitable for single-crystal X-ray diffraction analysis were obtained.

## Refinement

Crystal data, data collection and structure refinement details are summarized in Table 2 ▸. The Flack parameter was 0.29 (7), determined using 1297 quotients of the type [(*I*^+^) − (*I*^−^)]/[(*I*^+^) + (*I*^−^)] (Parsons *et al.*, 2013 ▸). Since the crystal contains both enanti­omers, this parameter concerns the polarity of the noncentrosymmetric crystal rather than the assignment of the mol­ecular absolute configurations.

## Supplementary Material

Crystal structure: contains datablock(s) I. DOI: 10.1107/S2414314626008588/vm4081sup1.cif

Structure factors: contains datablock(s) I. DOI: 10.1107/S2414314626008588/vm4081Isup2.hkl

Point-by-point response to the Co-editor' s comments for manuscript vm4081. DOI: 10.1107/S2414314626008588/vm4081sup3.docx

CCDC reference: 2582017

Additional supporting information:  crystallographic information; 3D view; checkCIF report

## Figures and Tables

**Table 1:** Hydrogen-bond geometry (Å, °)

*D*—H⋯*A*	*D*—H	H⋯*A*	*D*⋯*A*	*D*—H⋯*A*
O11—H11⋯O13^i^	0.82	2.00	2.783 (4)	160
O13—H13⋯O24^ii^	0.82	1.94	2.748 (4)	167
O23—H23⋯O14	0.82	2.00	2.805 (4)	169
O24—H24⋯O26^iii^	0.82	2.14	2.924 (4)	160
O25—H25⋯O22^iv^	0.82	2.27	2.862 (4)	129

Symmetry codes: (i) 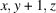; (ii) 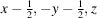; (iii) 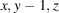; (iv) 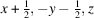.

**Table 2:** Experimental details

Crystal data	
Chemical formula	C_7_H_14_O_6_
*M*_r_	194.18
Crystal system, space group	Orthorhombic, *Pna*2_1_
Temperature (K)	296
*a*, *b*, *c* (Å)	12.9744 (6), 6.1793 (3), 20.9927 (9)
*V* (Å^3^)	1683.06 (13)
*Z*	8
Radiation type	Cu *K*α
μ (mm^−1^)	1.17
Crystal size (mm)	0.10 × 0.10 × 0.10

Data collection	
Diffractometer	Rigaku R-AXIS RAPID
Absorption correction	Multi-scan (*ABSCOR*; Rigaku, 1995 ▸)
*T*_min_, *T*_max_	0.639, 1.000
No. of measured, independent and observed [*I* > 2σ(*I*)] reflections	16861, 3055, 2873
*R*_int_	0.060
(sin θ/λ)_max_ (Å^−1^)	0.602

Refinement	
*R*[*F*^2^ > 2σ(*F*^2^)], *wR*(*F*^2^), *S*	0.048, 0.134, 1.06
No. of reflections	3055
No. of parameters	245
No. of restraints	1
H-atom treatment	H-atom parameters constrained
Δρ_max_, Δρ_min_ (e Å^−3^)	0.59, −0.26
Absolute structure	Flack *x* determined using 1297 quotients [(*I*^+^)−(*I*^−^)]/[(*I*^+^)+(*I*^−^)] (Parsons *et al.*, 2013 ▸)
Absolute structure parameter	0.29 (7)

Computer programs: *RAPID-AUTO* (Rigaku, 2009 ▸), *SHELXT2018/2* (Sheldrick, 2015*a* ▸), *SHELXL2018/3* (Sheldrick, 2015*b* ▸) and *OLEX2* (Dolomanov *et al.*, 2009 ▸).

## full crystallographic data

### Methyl α-D,L-sorboside Crystal data

**Table:**

C_7_H_14_O_6_	*D*_x_ = 1.533 Mg m^−^^3^
*M**_r_* = 194.18	Cu *K*α radiation, λ = 1.54187 Å
Orthorhombic, *P**n**a*2_1_	Cell parameters from 3106 reflections
*a* = 12.9744 (6) Å	θ = 4.4–68.2°
*b* = 6.1793 (3) Å	µ = 1.17 mm^−^^1^
*c* = 20.9927 (9) Å	*T* = 296 K
*V* = 1683.06 (13) Å^3^	Block, clear light colourless
*Z* = 8	0.1 × 0.1 × 0.1 mm
*F*(000) = 832	

### Methyl α-D,L-sorboside Data collection

**Table:**

Rigaku R-AXIS RAPID diffractometer	2873 reflections with *I* > 2σ(*I*)
ω scans	*R*_int_ = 0.060
Absorption correction: multi-scan (ABSCOR; Rigaku, 1995)	θ_max_ = 68.2°, θ_min_ = 4.2°
*T*_min_ = 0.639, *T*_max_ = 1.000	*h* = −14→15
16861 measured reflections	*k* = −7→7
3055 independent reflections	*l* = −25→25

### Methyl α-D,L-sorboside Refinement

**Table:**

Refinement on *F*^2^	Hydrogen site location: inferred from neighbouring sites
Least-squares matrix: full	H-atom parameters constrained
*R*[*F*^2^ > 2σ(*F*^2^)] = 0.048	*w* = 1/[σ^2^(*F*_o_^2^) + (0.1016*P*)^2^ + 0.0448*P*] where *P* = (*F*_o_^2^ + 2*F*_c_^2^)/3
*wR*(*F*^2^) = 0.134	(Δ/σ)_max_ = 0.005
*S* = 1.06	Δρ_max_ = 0.59 e Å^−^^3^
3055 reflections	Δρ_min_ = −0.25 e Å^−^^3^
245 parameters	Absolute structure: Flack *x* determined using 1297 quotients [(*I*^+^)-(*I*^-^)]/[(*I*^+^)+(*I*^-^)] (Parsons *et al.*, 2013)
1 restraint	Absolute structure parameter: 0.29 (7)
Primary atom site location: iterative	

### Methyl α-D,L-sorboside Special details

**Table:**

Geometry. All esds (except the esd in the dihedral angle between two l.s. planes) are estimated using the full covariance matrix. The cell esds are taken into account individually in the estimation of esds in distances, angles and torsion angles; correlations between esds in cell parameters are only used when they are defined by crystal symmetry. An approximate (isotropic) treatment of cell esds is used for estimating esds involving l.s. planes.
Refinement. The structure was solved using SHELXT (Sheldrick, 2015*a*) and refined against F^2^ by full-matrix least-squares methods using SHELXL (Sheldrick, 2015*b*). All non-hydrogen atoms were refined anisotropically. All H atoms were placed in calculated positions and refined using constrained models. C-bound H atoms were refined using riding models, with C—H = 0.96–0.98 Å and U_iso_(H) = 1.2U_eq_(C), or 1.5U_eq_(C) for methyl groups. Hydroxy H atoms were treated as rotating groups, with O—H = 0.82 Å and U_iso_(H) = 1.5U_eq_(O). The methyl groups at C17 and C27 were refined as rotating groups using AFIX 137. The final refinement gave R_1_ = 0.0481 for reflections with I > 2σ(I) and wR_2_ = 0.1342 for all data. The maximum and minimum residual electron densities were 0.59 and -0.26 e Å^-3^, respectively.

### Methyl α-D,L-sorboside Fractional atomic coordinates and isotropic or equivalent isotropic displacement parameters (Å^2^)

**Table:**

	*x*	*y*	*z*	*U*_iso_*/*U*_eq_	
O11	−0.4003 (3)	0.3240 (5)	−0.78069 (16)	0.0671 (10)	
H11	−0.387471	0.451934	−0.773944	0.101*	
O12	−0.4126 (2)	−0.0201 (4)	−0.64021 (12)	0.0396 (6)	
O13	−0.4084 (2)	−0.2354 (4)	−0.75361 (13)	0.0410 (6)	
H13	−0.436954	−0.186441	−0.785323	0.062*	
O14	−0.1999 (2)	−0.3795 (4)	−0.74496 (14)	0.0480 (7)	
H14	−0.232748	−0.492633	−0.743066	0.072*	
O15	−0.0851 (3)	−0.1770 (7)	−0.64158 (15)	0.0647 (10)	
H15	−0.044367	−0.244186	−0.663935	0.097*	
O16	−0.2831 (2)	0.2182 (4)	−0.67377 (12)	0.0392 (6)	
C11	−0.4427 (3)	0.2310 (6)	−0.72577 (19)	0.0420 (9)	
H11A	−0.503304	0.147803	−0.737335	0.050*	
H11B	−0.464228	0.344856	−0.696879	0.050*	
C12	−0.3666 (3)	0.0849 (5)	−0.69220 (16)	0.0303 (7)	
C13	−0.3273 (3)	−0.0967 (5)	−0.73539 (14)	0.0302 (7)	
H13A	−0.298012	−0.031393	−0.773888	0.036*	
C14	−0.2434 (3)	−0.2254 (5)	−0.70227 (15)	0.0334 (7)	
H14A	−0.272427	−0.300395	−0.665259	0.040*	
C15	−0.1593 (3)	−0.0737 (6)	−0.68080 (16)	0.0401 (8)	
H15A	−0.124857	−0.012794	−0.718270	0.048*	
C16	−0.2033 (3)	0.1069 (7)	−0.6415 (2)	0.0461 (9)	
H16A	−0.230163	0.048194	−0.601969	0.055*	
H16B	−0.148858	0.208235	−0.630911	0.055*	
C17	−0.4440 (4)	0.1097 (8)	−0.5876 (2)	0.0565 (11)	
H17A	−0.463447	0.250764	−0.602523	0.085*	
H17B	−0.387895	0.123021	−0.557999	0.085*	
H17C	−0.501660	0.042948	−0.566790	0.085*	
O21	−0.1728 (3)	0.2626 (6)	−0.83033 (15)	0.0693 (10)	
H21	−0.121087	0.338497	−0.832471	0.104*	
O22	−0.16767 (18)	−0.0279 (4)	−0.98010 (10)	0.0352 (5)	
O23	−0.1854 (2)	−0.2583 (4)	−0.87343 (12)	0.0432 (6)	
H23	−0.184412	−0.307650	−0.837178	0.065*	
O24	0.0240 (3)	−0.4150 (5)	−0.86951 (14)	0.0574 (8)	
H24	0.021034	−0.534701	−0.886293	0.086*	
O25	0.1493 (3)	−0.2152 (8)	−0.97162 (17)	0.0809 (13)	
H25	0.197986	−0.259479	−0.950410	0.121*	
O26	−0.04060 (18)	0.1940 (4)	−0.93706 (13)	0.0391 (6)	
C21	−0.2054 (3)	0.2084 (6)	−0.8919 (2)	0.0443 (9)	
H21A	−0.214372	0.339403	−0.916781	0.053*	
H21B	−0.271419	0.135103	−0.889677	0.053*	
C22	−0.1280 (2)	0.0639 (5)	−0.92409 (15)	0.0295 (7)	
C23	−0.0973 (3)	−0.1279 (5)	−0.88316 (15)	0.0313 (7)	
H23A	−0.073788	−0.073839	−0.841740	0.038*	
C24	−0.0105 (3)	−0.2554 (6)	−0.91335 (17)	0.0373 (8)	
H24A	−0.036453	−0.327448	−0.951715	0.045*	
C25	0.0787 (3)	−0.1094 (7)	−0.9316 (2)	0.0471 (9)	
H25A	0.114018	−0.059367	−0.893078	0.057*	
C26	0.0401 (3)	0.0828 (7)	−0.9690 (2)	0.0481 (9)	
H26A	0.096793	0.182073	−0.976239	0.058*	
H26B	0.015408	0.033996	−1.010144	0.058*	
C27	−0.1993 (4)	0.1151 (7)	−1.0295 (2)	0.0519 (10)	
H27A	−0.250429	0.045299	−1.055461	0.078*	
H27B	−0.140919	0.152506	−1.055372	0.078*	
H27C	−0.227987	0.243990	−1.011148	0.078*	

### Methyl α-D,L-sorboside Atomic displacement parameters (Å^2^)

**Table:**

	*U*^11^	*U*^22^	*U*^33^	*U*^12^	*U*^13^	*U*^23^
O11	0.103 (3)	0.0426 (15)	0.0562 (17)	0.0166 (18)	−0.0040 (18)	0.0075 (14)
O12	0.0445 (14)	0.0373 (13)	0.0370 (11)	−0.0016 (11)	0.0099 (10)	−0.0032 (10)
O13	0.0477 (15)	0.0299 (12)	0.0454 (13)	−0.0059 (10)	−0.0151 (11)	−0.0024 (10)
O14	0.0561 (17)	0.0349 (13)	0.0529 (15)	0.0097 (12)	0.0092 (13)	−0.0099 (11)
O15	0.0482 (18)	0.090 (3)	0.0559 (17)	0.0319 (17)	−0.0164 (14)	−0.0109 (17)
O16	0.0336 (13)	0.0310 (11)	0.0529 (14)	−0.0064 (10)	−0.0059 (11)	−0.0074 (10)
C11	0.040 (2)	0.0313 (17)	0.054 (2)	0.0046 (15)	−0.0051 (17)	0.0005 (14)
C12	0.0278 (16)	0.0275 (16)	0.0356 (15)	−0.0033 (12)	−0.0015 (13)	−0.0033 (12)
C13	0.0364 (18)	0.0258 (14)	0.0285 (14)	−0.0027 (13)	−0.0019 (12)	−0.0001 (11)
C14	0.0387 (19)	0.0325 (16)	0.0290 (14)	0.0071 (14)	0.0067 (14)	−0.0001 (12)
C15	0.0277 (18)	0.051 (2)	0.0416 (19)	0.0050 (15)	−0.0044 (13)	−0.0019 (14)
C16	0.032 (2)	0.052 (2)	0.055 (2)	0.0016 (17)	−0.0107 (16)	−0.0136 (16)
C17	0.055 (3)	0.068 (3)	0.047 (2)	0.016 (2)	0.0114 (19)	−0.012 (2)
O21	0.092 (3)	0.0563 (19)	0.0600 (19)	0.0102 (18)	0.0099 (18)	−0.0186 (15)
O22	0.0382 (12)	0.0340 (11)	0.0335 (11)	−0.0021 (10)	−0.0099 (10)	0.0036 (10)
O23	0.0476 (14)	0.0363 (13)	0.0457 (14)	−0.0136 (11)	−0.0039 (12)	0.0085 (11)
O24	0.079 (2)	0.0365 (14)	0.0570 (16)	0.0221 (14)	−0.0342 (15)	−0.0060 (12)
O25	0.051 (2)	0.128 (3)	0.063 (2)	0.050 (2)	−0.0025 (17)	−0.013 (2)
O26	0.0336 (13)	0.0321 (11)	0.0515 (13)	−0.0043 (10)	0.0045 (11)	0.0018 (10)
C21	0.041 (2)	0.038 (2)	0.054 (2)	0.0077 (15)	0.0128 (17)	−0.0017 (15)
C22	0.0285 (16)	0.0250 (14)	0.0351 (15)	−0.0041 (12)	−0.0003 (12)	−0.0006 (12)
C23	0.0353 (18)	0.0281 (16)	0.0306 (15)	−0.0029 (13)	−0.0035 (13)	−0.0007 (12)
C24	0.043 (2)	0.0344 (16)	0.0349 (15)	0.0111 (14)	−0.0144 (15)	−0.0068 (13)
C25	0.0335 (19)	0.066 (3)	0.0417 (17)	0.0126 (17)	−0.0028 (16)	−0.0048 (17)
C26	0.0255 (18)	0.067 (3)	0.052 (2)	0.0012 (16)	0.0050 (16)	0.0044 (18)
C27	0.051 (2)	0.061 (3)	0.044 (2)	0.012 (2)	−0.0096 (17)	0.0136 (18)

### Methyl α-D,L-sorboside Geometric parameters (Å, º)

**Table:**

O11—H11	0.8200	O21—H21	0.8200
O11—C11	1.401 (5)	O21—C21	1.401 (5)
O12—C12	1.403 (4)	O22—C22	1.403 (4)
O12—C17	1.425 (4)	O22—C27	1.424 (4)
O13—H13	0.8200	O23—H23	0.8200
O13—C13	1.410 (4)	O23—C23	1.414 (4)
O14—H14	0.8200	O24—H24	0.8200
O14—C14	1.424 (4)	O24—C24	1.421 (4)
O15—H15	0.8200	O25—H25	0.8200
O15—C15	1.418 (4)	O25—C25	1.404 (5)
O16—C12	1.415 (4)	O26—C22	1.417 (4)
O16—C16	1.416 (5)	O26—C26	1.420 (4)
C11—H11A	0.9700	C21—H21A	0.9700
C11—H11B	0.9700	C21—H21B	0.9700
C11—C12	1.513 (5)	C21—C22	1.504 (5)
C12—C13	1.530 (4)	C22—C23	1.518 (4)
C13—H13A	0.9800	C23—H23A	0.9800
C13—C14	1.517 (4)	C23—C24	1.513 (5)
C14—H14A	0.9800	C24—H24A	0.9800
C14—C15	1.507 (5)	C24—C25	1.516 (6)
C15—H15A	0.9800	C25—H25A	0.9800
C15—C16	1.501 (5)	C25—C26	1.509 (6)
C16—H16A	0.9700	C26—H26A	0.9700
C16—H16B	0.9700	C26—H26B	0.9700
C17—H17A	0.9600	C27—H27A	0.9600
C17—H17B	0.9600	C27—H27B	0.9600
C17—H17C	0.9600	C27—H27C	0.9600
			
C11—O11—H11	109.5	C21—O21—H21	109.5
C12—O12—C17	117.7 (3)	C22—O22—C27	117.7 (3)
C13—O13—H13	109.5	C23—O23—H23	109.5
C14—O14—H14	109.5	C24—O24—H24	109.5
C15—O15—H15	109.5	C25—O25—H25	109.5
C12—O16—C16	114.0 (3)	C22—O26—C26	114.0 (3)
O11—C11—H11A	109.2	O21—C21—H21A	109.5
O11—C11—H11B	109.2	O21—C21—H21B	109.5
O11—C11—C12	111.8 (3)	O21—C21—C22	110.8 (3)
H11A—C11—H11B	107.9	H21A—C21—H21B	108.1
C12—C11—H11A	109.2	C22—C21—H21A	109.5
C12—C11—H11B	109.2	C22—C21—H21B	109.5
O12—C12—O16	112.5 (3)	O22—C22—O26	111.2 (3)
O12—C12—C11	111.1 (3)	O22—C22—C21	111.8 (3)
O12—C12—C13	105.3 (3)	O22—C22—C23	104.8 (2)
O16—C12—C11	106.2 (3)	O26—C22—C21	106.5 (3)
O16—C12—C13	109.5 (3)	O26—C22—C23	110.0 (3)
C11—C12—C13	112.3 (3)	C21—C22—C23	112.6 (3)
O13—C13—C12	111.0 (3)	O23—C23—C22	108.3 (3)
O13—C13—H13A	108.4	O23—C23—H23A	108.5
O13—C13—C14	109.9 (3)	O23—C23—C24	111.5 (3)
C12—C13—H13A	108.4	C22—C23—H23A	108.5
C14—C13—C12	110.6 (3)	C24—C23—C22	111.4 (3)
C14—C13—H13A	108.4	C24—C23—H23A	108.5
O14—C14—C13	110.3 (3)	O24—C24—C23	108.9 (3)
O14—C14—H14A	109.6	O24—C24—H24A	109.0
O14—C14—C15	108.5 (3)	O24—C24—C25	109.7 (3)
C13—C14—H14A	109.6	C23—C24—H24A	109.0
C15—C14—C13	109.3 (3)	C23—C24—C25	111.4 (3)
C15—C14—H14A	109.6	C25—C24—H24A	109.0
O15—C15—C14	112.6 (3)	O25—C25—C24	111.9 (4)
O15—C15—H15A	109.2	O25—C25—H25A	109.6
O15—C15—C16	105.9 (3)	O25—C25—C26	105.8 (3)
C14—C15—H15A	109.2	C24—C25—H25A	109.6
C16—C15—C14	110.6 (3)	C26—C25—C24	110.3 (3)
C16—C15—H15A	109.2	C26—C25—H25A	109.6
O16—C16—C15	112.1 (3)	O26—C26—C25	112.4 (3)
O16—C16—H16A	109.2	O26—C26—H26A	109.1
O16—C16—H16B	109.2	O26—C26—H26B	109.1
C15—C16—H16A	109.2	C25—C26—H26A	109.1
C15—C16—H16B	109.2	C25—C26—H26B	109.1
H16A—C16—H16B	107.9	H26A—C26—H26B	107.9
O12—C17—H17A	109.5	O22—C27—H27A	109.5
O12—C17—H17B	109.5	O22—C27—H27B	109.5
O12—C17—H17C	109.5	O22—C27—H27C	109.5
H17A—C17—H17B	109.5	H27A—C27—H27B	109.5
H17A—C17—H17C	109.5	H27A—C27—H27C	109.5
H17B—C17—H17C	109.5	H27B—C27—H27C	109.5
			
O11—C11—C12—O12	−175.4 (3)	O21—C21—C22—O22	167.9 (3)
O11—C11—C12—O16	62.0 (4)	O21—C21—C22—O26	−70.4 (4)
O11—C11—C12—C13	−57.7 (4)	O21—C21—C22—C23	50.2 (4)
O12—C12—C13—O13	57.7 (3)	O22—C22—C23—O23	−58.0 (3)
O12—C12—C13—C14	−64.7 (3)	O22—C22—C23—C24	64.9 (3)
O13—C13—C14—O14	63.0 (4)	O23—C23—C24—O24	−66.3 (4)
O13—C13—C14—C15	−177.8 (3)	O23—C23—C24—C25	172.6 (3)
O14—C14—C15—O15	−68.1 (4)	O24—C24—C25—O25	72.3 (4)
O14—C14—C15—C16	173.7 (3)	O24—C24—C25—C26	−170.3 (3)
O15—C15—C16—O16	−176.9 (3)	O25—C25—C26—O26	173.8 (3)
O16—C12—C13—O13	178.8 (3)	O26—C22—C23—O23	−177.6 (3)
O16—C12—C13—C14	56.5 (4)	O26—C22—C23—C24	−54.7 (3)
C11—C12—C13—O13	−63.4 (3)	C21—C22—C23—O23	63.8 (4)
C11—C12—C13—C14	174.3 (3)	C21—C22—C23—C24	−173.3 (3)
C12—O16—C16—C15	58.2 (4)	C22—O26—C26—C25	−59.0 (4)
C12—C13—C14—O14	−174.0 (3)	C22—C23—C24—O24	172.5 (3)
C12—C13—C14—C15	−54.9 (3)	C22—C23—C24—C25	51.5 (4)
C13—C14—C15—O15	171.6 (3)	C23—C24—C25—O25	−167.1 (3)
C13—C14—C15—C16	53.4 (4)	C23—C24—C25—C26	−49.7 (4)
C14—C15—C16—O16	−54.6 (4)	C24—C25—C26—O26	52.7 (5)
C16—O16—C12—O12	58.4 (4)	C26—O26—C22—O22	−56.7 (4)
C16—O16—C12—C11	−179.8 (3)	C26—O26—C22—C21	−178.8 (3)
C16—O16—C12—C13	−58.3 (4)	C26—O26—C22—C23	58.9 (4)
C17—O12—C12—O16	52.4 (4)	C27—O22—C22—O26	−58.6 (4)
C17—O12—C12—C11	−66.5 (4)	C27—O22—C22—C21	60.3 (4)
C17—O12—C12—C13	171.7 (3)	C27—O22—C22—C23	−177.4 (3)

### Methyl α-D,L-sorboside Hydrogen-bond geometry (Å, º)

**Table:**

*D*—H···*A*	*D*—H	H···*A*	*D*···*A*	*D*—H···*A*
O11—H11···O13^i^	0.82	2.00	2.783 (4)	160
O13—H13···O24^ii^	0.82	1.94	2.748 (4)	167
O23—H23···O14	0.82	2.00	2.805 (4)	169
O24—H24···O26^iii^	0.82	2.14	2.924 (4)	160
O25—H25···O22^iv^	0.82	2.27	2.862 (4)	129

Symmetry codes: (i) *x*, *y*+1, *z*; (ii) *x*−1/2, −*y*−1/2, *z*; (iii) *x*, *y*−1, *z*; (iv) *x*+1/2, −*y*−1/2, *z*.
