# Supplementary material for: Methyl α-d,l-sorboside
Source: IUCrdata. 2026 Aug 20;11(Pt 8):x260858. doi: 10.1107/S2414314626008588 (PMC13548381; doi:10.1107/S2414314626008588)
Supplement: Supplementary file 1 [file x-11-x260858-sup3.docx]

Response to Co-editor Professor L. Van Meervelt

Title: Methyl α-D,L-sorboside

Manuscript ID: vm4081

CCDC deposition number: 2577689

Dear Professor Van Meervelt,

We sincerely thank you for your careful assessment of our data article and for your helpful comments. We have revised the structural model, manuscript, Table 2, Figure 1 and Figure 2 in accordance with your suggestions. Our point-by-point responses are provided below.

The crystallographic data for the title compound have been deposited with the Cambridge Crystallographic Data Centre under CCDC deposition number 2577689.

**Comment 1:**

The geometry of methyl groups C27 (angles between 82.9 and 119.4 degrees) and C17 (102.1–117.3 degrees) is not OK after free refinement of the H atoms. Refine both methyl groups as rotating groups (AFIX 137).

**Response:**

The methyl groups at C17 and C27 have been re-refined as rotating groups using AFIX 137. Both methyl groups now have idealized tetrahedral geometry, with the corresponding H—C—H and O—C—H angles equal to 109.5°. The revised refinement converged to R1 = 0.0481 for reflections with I > 2σ(I) and wR2 = 0.1342 for all data. The CIF and the refinement description have been updated accordingly.

**Comment 2:**

Line 23: add also the axial position of the -OMe substituent.

**Response:**

The axial orientation of the methoxy substituents has been added to the structural description. The relevant passage has been revised as follows:

“The D and L enantiomers adopt mirror-related ⁵C₂ and ²C₅ chair conformations, respectively. In both molecules, the methoxy substituent at the anomeric carbon atom (C12 or C22) occupies an axial position.”

**Comment 3:**

Line 35: give ratio of D- and L-sorbose in mixture.

**Response:**

The starting materials were mixed in an equimolar ratio. The D:L ratio of 1:1 has now been stated explicitly in the synthesis section. The synopsis and abstract have also been revised to include this information.

**Comment 4:**

Line 44: modify treatment of hydrogen atoms refined freely – also H23A, H24A, H25A and H14 were refined freely, but what is the added value?

**Response:**

The hydrogen-atom treatment has been revised. Free refinement of the H atoms offered no clear crystallographic advantage and was therefore discontinued. All H atoms are now refined using constrained models. C-bound H atoms, including H23A, H24A and H25A, were placed in calculated positions and refined using riding models. The hydroxy H atom H14, together with the other hydroxy H atoms, was treated as part of a rotating group. The methyl groups at C17 and C27 were refined as rotating groups using AFIX 137. The refinement section and the corresponding CIF items have been revised accordingly.

**Comment 5:**

Figure 1: no need to label the H atoms, add O23-H23…O14 hydrogen bond between both molecules in the asymmetric unit.

**Response:**

Figure 1 has been replaced with a revised ORTEP drawing. The H-atom labels have been removed, and the O23—H23···O14 hydrogen bond linking the two crystallographically independent molecules in the asymmetric unit is now shown as a dashed line. The Figure 1 caption has also been revised accordingly.

Comment 6:

Figure 2: add indication unit cell directions or unit cell.

**Response:**

Figure 2 has been revised to include the a-, b- and c-axis directions. The Figure 2 caption has also been revised to state that the crystallographic axis directions are indicated.

**Comment 7:**

Table 2: add missing O24-H24…O26 hydrogen bond (also in Figure 2).

**Response:**

The missing O24—H24···O26 hydrogen bond has been added to Table 2, with the following geometry: O—H = 0.82 Å, H···O = 2.14 Å, O···O = 2.924(4) Å and O—H···O = 160°. This interaction is also shown as a cyan dashed line in the revised Figure 2. The structural discussion has been updated to describe the five crystallographically distinct O—H···O hydrogen bonds listed in Table 2.

We hope that these revisions have satisfactorily addressed all of the comments and that the revised data article is now suitable for publication in IUCrData.

Yours sincerely,

Tomoko Takashita

Graduate School of Science for Creative Emergence, Kagawa University, Japan
